# Current status of using adsorbent nanomaterials for removing microplastics from water supply systems: a mini review

**DOI:** 10.3762/bjnano.16.127

**Published:** 2025-10-21

**Authors:** Nguyen Thi Nhan, Tran Le Luu

**Affiliations:** 1 Master Program in Water Technology, Reuse and Management, Vietnamese German University, Ho Chi Minh City, Vietnam

**Keywords:** adsorbent interactions, adsorbent nanomaterials, carbon-based adsorbents, metal-organic frameworks (MOFs), microplastics (MPs)

## Abstract

The widespread use of plastic has led to microplastics (MPs) being released in many water sources. MP contamination in water supply systems is a global concern due to their persistence and ability to adsorb toxic pollutants. Despite having effectiveness, conventional water treatment processes still have limited efficiency in removing MPs, especially smaller particles. Thus, it requires researchers to develop effective and sustainable strategies to deal with this matter. Many studies have shown that adsorbent nanomaterials have potential for the removal of MPs from water. This review evaluates the current status of using adsorbent nanomaterials in removing MPs from water supply systems. It discusses the occurrences and removal efficiency of MPs in water supply systems, as well as the mechanisms and performance when applying these materials for treatment. In addition, the related risk of adsorbent nanomaterials is also considered. Microplastics from land-based sources and wastewater plants persist in water supplies, with conventional treatments removing only 40–70%, especially struggling with smaller particles. Based mainly on mechanisms like electrostatic interactions, hydrophobic interactions, pore filling, hydrogen bonding, π–π stacking, and surface complexation, adsorbent nanomaterials achieve over 90% removal of MPs and can recovery. Their effectiveness depends on material properties and environmental factors, but challenges remain in scale-up and related risks. Adsorbent nanomaterials show promising potential to enhance MP removal through specific properties. Although some related risks are discussed, these materials provide a foundation for developing sustainable, effective solutions to mitigate MPs pollution in the water supply system.

## Introduction

Plastic materials have become an indispensable part of modern society because of their distinct characteristics, such as low production cost, significant durability, and high flexibility. Global plastic production has risen dramatically over the past decades, reaching approximately 288 million tonnes annually, and it is projected to rise to 33,000 million tonnes by 2050 [1]. However, despite this significant increase in production, the global recycling rate remaines low at approximately 9% since 1950, resulting in the accumulation of plastic waste in ecological and environmental systems [2–3].

The issues of microplastics (MPs) related to public health and environmental risks have gained significant attention [1]. Because of their small size, high surface area, and hydrophobic properties, MPs can act as vectors for toxic chemicals, including heavy metals (lead, cadmium, or mercury) and persistent organic pollutants like polychlorinated biphenyls, polycyclic aromatic hydrocarbons, and dichlorodiphenyltrichloroethane [4–6]. These adsorbed contaminants can bioaccumulate through the food chain and move from marine organisms to human food [7]. In water supply systems, MPs with adsorbed toxins pose significant risks without being properly removed during treatment [8]. Exposure to MPs and their adsorbed contaminants through water supply systems has been related to various adverse health effects, including endocrine disruption, neurotoxicity, carcinogenesis, and chronic exposure-related issues [9–10]. Ecologically, MPs disrupt aquatic ecosystems by interfering with feeding patterns (reduced ingestion rates), reproduction (lower egg production and fertilization success), and growth rates (lack of energy and tissue damage) in marine organisms [11–12]. Thus, the persistence of MPs threatens not only biodiversity and ecosystem stability but also human health over the long term.

These risks highlight the urgent need for effective strategies to mitigate MP pollution in both environmental and water supply systems. While numerous review papers have been conducted to evaluate the status of MP pollution, most of these papers have focused separately on sources and occurrences in the natural environment [13–15], contaminant interactions [16–17], risk assessments [18–19], extraction and analysis methods [20–21], and removal technologies of MPs [22–23] without focusing on water supply systems, which directly affect human daily life, and the potential application of adsorbent nanomaterials for MP removal. Sajid et al. provided an overview of various adsorbent materials and their efficiency [24]. However, the authors do not deeply explore the potential challenges related to large-scale applications or the integration of these materials into existing water treatment systems. Similarly, the reviews by Yu et al. and Das et al. highlighted the purification potential of different nanomaterials but lack a detailed discussion on the risks and limitations of these materials, particularly in the context of water supply systems [25–26].

To deal with current gaps, this review aims to provide aspects relating to (i) the occurrences of MPs in water supply systems and the effectiveness of MP removal throughout the treatment processes; (ii) the potential of adsorbent nanomaterials for MP removal, focusing on adsorption mechanisms and performance; and (iii) risk assessments and associated problems when applying adsorbent nanomaterials. In addition, it is important to identify critical gaps regarding large-scale applications and insufficient integration into existing systems. By expanding the scope of the research to evaluate the current status of adsorbent nanomaterials’ applicability and risks for removing MPs from water supply systems, this review differs from others. Addressing these gaps is essential for developing sustainable solutions that can effectively mitigate MP pollution in water supply systems while protecting both human health and aquatic ecosystems.

## Review

### Sources and distribution pathways of MPs to water supply system

**Figure 1:**
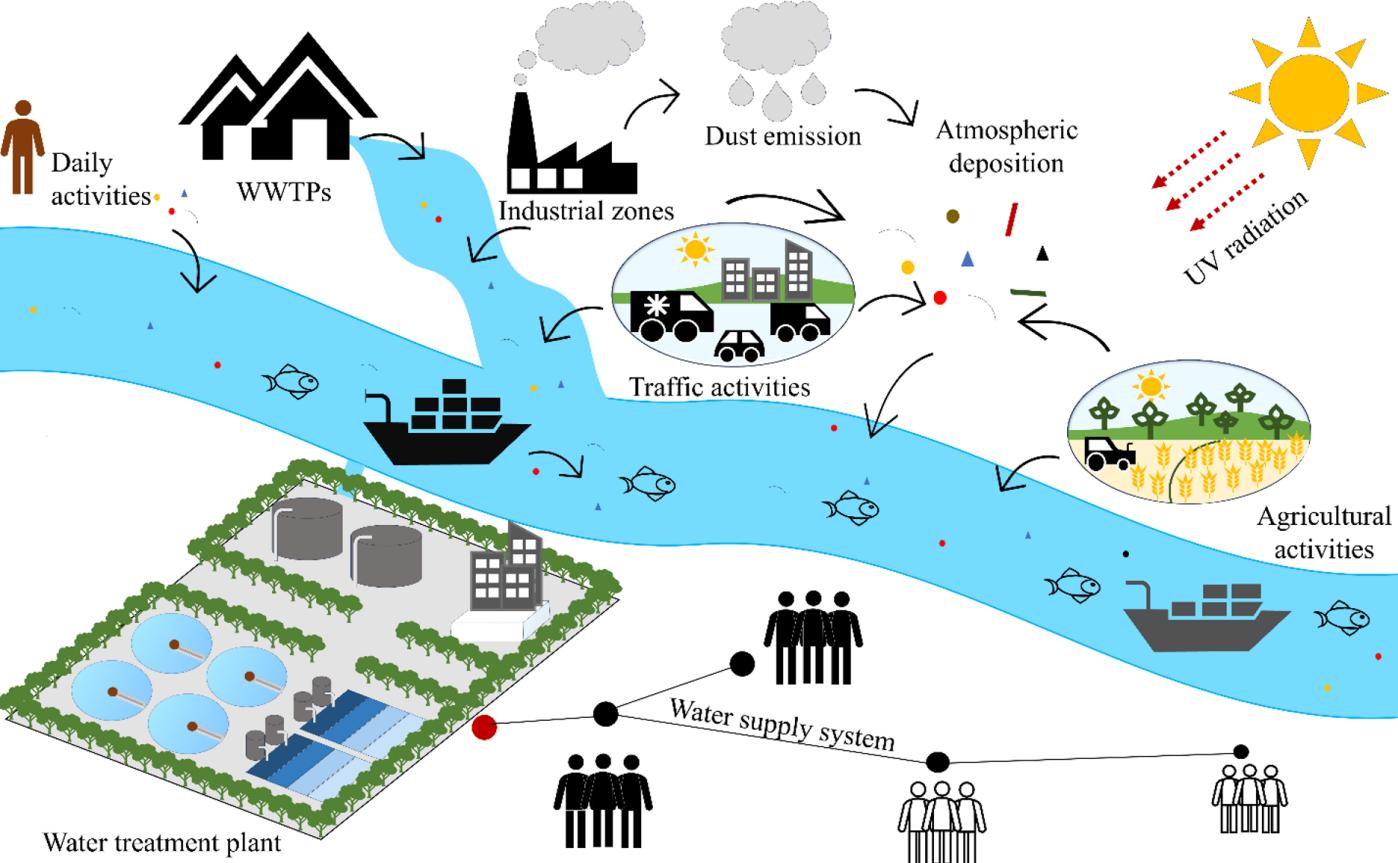
Sources and distribution of MPs in environmental systems.

Microplastics can be classified as primary or secondary. Primary MPs are intentionally manufactured for various applications, whereas secondary MPs result from the degradation or breakdown of plastic waste by physical, chemical, or biological factors [27]. Figure 1 illustrates the sources and distribution of MPs from the natural environment to water supply systems. According to the study of Osman et al., land-based sources originating from plastic bags, bottles, personal care products, construction materials, clothing, sewage sludge, urban runoff, and industrial activities contribute 80–90% of MPs in water bodies [28]. The outputs from wastewater treatment plants (WWTPs) are identified as a major pathway for MP discharge into aquatic environments [29]. Data collected worldwide shows that millions of MPs are still being released. In Türkiye, a total of 6.18 × 10^10^ MPs from 15 WWTPs investigated were discharged into the marine environment per day [30]. A number of 4.95 × 10^4^ to 1.49 × 10^8^ MPs entering the environment daily was recorded in six WWTPs in Iran [31]. A study in Morocco demonstrated a significant amount of MPs discharged into the marine environment, with a daily average ranging from 1.6 × 10^8^ MPs per day to 9.9 × 10^8^ MPs per day in the summer [32]. Ocean-based sources, such as tourism, fishing, aquaculture, and maritime industries, account for the remaining 10–20% of MPs released into water bodies. An estimated 4622 t of MPs from commercial fishing-related activities, generated by fishing gear, nets, and ropes, are produced per year [33]. In terms of the maritime industry, the study found that new and one-year-old ropes released fewer microplastics (14–22 fragments, 11–12 μg·m^−1^) compared to two-year-old (720–247 μg·m^−1^) and ten-year-old ropes (767–1052 μg·m^−1^) [34]. Thus, natural water sources such as surface water (rivers, lakes, and streams) and groundwater have received large amounts of MPs from various sources. These water sources play an important role in water supply systems worldwide, and MP pollution directly affects the water quality used in water treatment plants (WTPs) [35].

### The occurrences of MPs in water supply systems

The water distribution systems are responsible for transporting treated water from WTPs to various locations through the distribution pipeline network. Depending on different characteristics, including the material of pipeline, distance of transportation, analytical methods, and the size of the MPs targeted, the concentration of MPs in water will fluctuate, as shown in Table 1. Research indicated that raw water sources (rivers and lakes) often contain higher levels of MPs because of direct contact with the environment, with concentrations ranging from 1473 to 3605 particles·L^−1^. After undergoing various treatment stages, these values decrease significantly, and MPs are still detected in treated water, with concentrations between 338 and 628 particles·L^−1^ [36]. Due to their small size and chemical stability, MPs can pass through conventional water treatment processes and infiltrate water supply systems. As reported in the study by Dalmau-Soler et al., 38% of drinking water samples from the supply network contained MPs (0.01 particles·L^−1^). Results suggest that some particles were related to maintenance activities, while pipes and infrastructure did not significantly contribute to MP contamination [37]. Many studies have reported different results, with MP concentrations in tap water ranging from 1 to 61,000 particles·L^−1^, in which the bulk of the data is approximately 569–751 particles·kg^−1^ [38–39]. In the study of Chu et al., MP concentrations in the water and pipe scale samples ranged from 13.2 to 134.7 particles·L^−1^ and from 569.9 to 751.7 particles·kg^−1^, respectively, with a significantly smaller particle size in the pipe scales (50–100 μm) than in the water samples (>200 μm) [40]. In Eastern China, the level of MPs in raw water was recorded at 4960 particles·L^−1^. After being treated by various processes, the MPs concentration significantly reduced from 4712 ± 95 particles·L^−1^ to 1012 ± 78 particles·L^−1^ [41]. Bottled water, which is normally considered safe, also contains MPs. In China, bottled water had MP levels between 13.6 and 39.3 particles·L^−1^ [42]. Research conducted in Malaysia shows that an average of 1421.20 ± 915.70 particles were found in one liter of bottled water, with most sizes below 50 µm [43]. In Bangladesh, different concentrations of MPs were found in water stored in various types of materials. The study indicated that water samples stored in glass bottles had the highest concentration of MPs (151 ± 14 particles·L^−1^), followed by cans (134 ± 14 particles·L^−1^), and PET bottles (95 ± 35 particles·L^−1^) [44]. Due to the incomplete removal of MPs by WTPs and their widespread presence in water supply systems, there is an urgent need for technologies that can effectively address this issue.

**Table 1:** An overview of the presence of MPs in the water supply system.

Location	Sample types	MP concentration	Size	Polymer types^a^	Ref.

China	water, pipe scale	water: 13.23–134.79 particles·L^−1^; pipe scale: 569.99–751.73 particles·kg^−1^	water: >200 μm; pipe scale: 50–100 μm	nylon, PVC	[40]
China	tap water, pipe scale	water: 1.74–20.88 particles·L^−1^; pipe scale: 0.03–3.48 particles·cm^−2^	water: 21–971 μm; pipe scale: 20–2055 μm	water: PA 70.3%; pipe scale: PET 50.0%	[45]
United Kingdom	tap water; bottled water	tap: 6–100 particles·L^−1^; bottled water: 12–62 particles·L^−1^	tap: 32.4 μm; bottled water: 26.5 μm	PP, PE, PVC, PET	[46]
Britain	tap water	0.017–0.1513 particles·L^−1^	>25 μm	19 polymers; PA, PET, PP, and PS commonly	[39]
China	tap water, bottled water	tap: 9.7–9.8 particles·L^−1^; bottled water: 13.6–39.3 particles·L^−1^	1–5000 μm	fragments, fibers	[42]
Flanders, Belgium	tap water	0.01 ± 0.02 particles·L^−1^	25–1000 μm	PP, PET	[47]
Europe	raw water; treated water	raw water: (1473 ± 34)–(3605 ± 497) particles·L^−1^; treated water: (338 ± 76)–(628 ± 28) particles·L^−1^	1–5000 μm	PE, PET, PP	[36]
İstanbul, Turkey	tap water	10–390 particles·L^−1^	12–4882 μm	EPP, NP, PE, PET, PP, PVC, PTA, VAC	[48]

^a^Ethylene propylene (EPP), neoprene (NP), polyethylene (PE), polyethylene terephthalate (PET), polypropylene (PP), polyvinyl chloride (PVC), polytetrafluoroethylene (PTA), vinyl chloride vinyl acetate copolymer (VAC), polyamide (PA), and polystyrene (PS).

### Potential, classification, and comparison of adsorbent nanomaterials and other treatment methods

**Figure 2:**
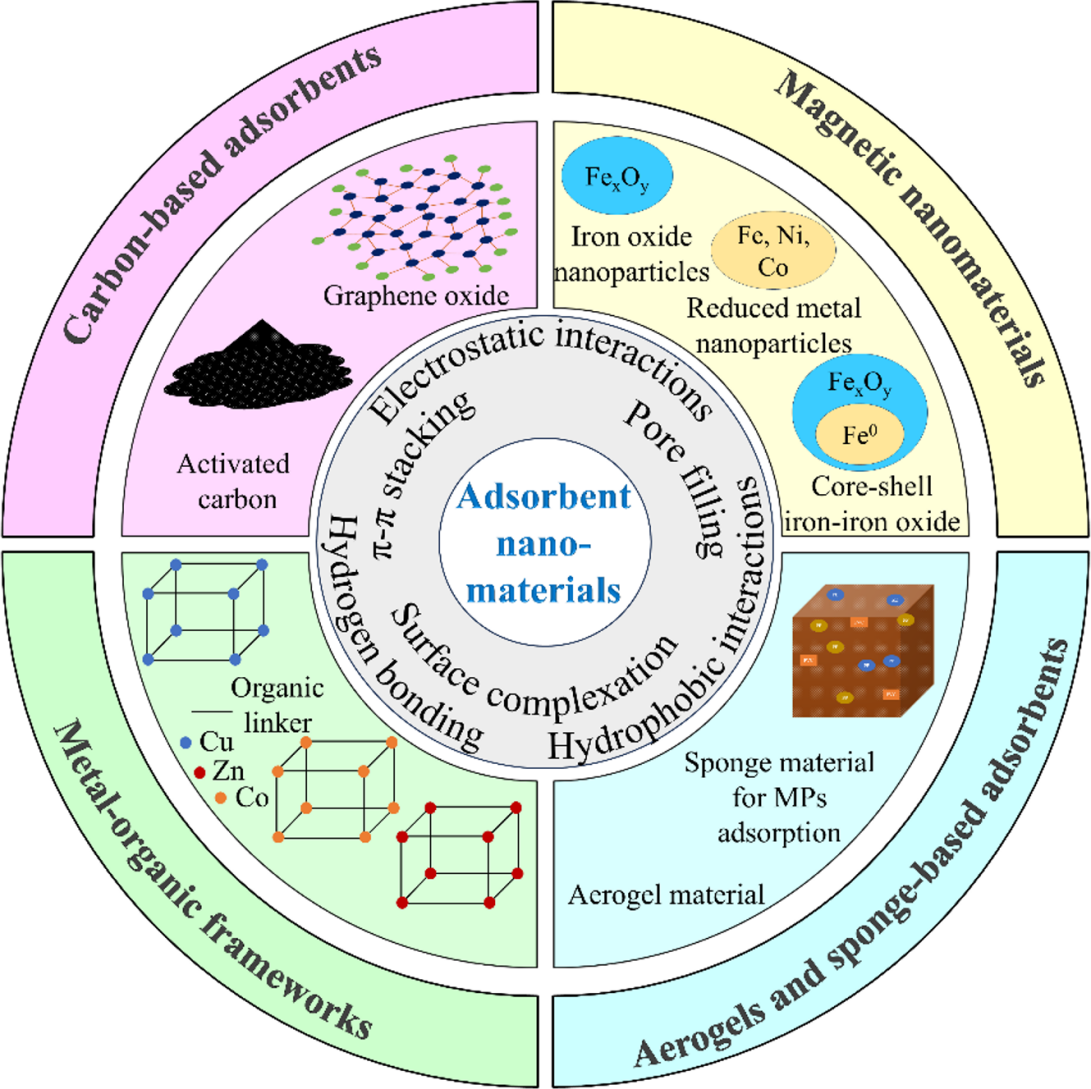
An illustration of the four main groups and mechanisms of adsorbent nanomaterials.

Adsorbent nanomaterials have recently shown great potential for removing MPs. They can be classified into four main groups, including carbon-based adsorbents, metal-organic frameworks (MOFs), magnetic nanomaterials, and aerogels and sponge-based adsorbents [49]. These materials are fabricated and modified to interact with different polymer compositions of MPs, such as polyethylene (PE), polypropylene (PP), and polystyrene (PS). The main mechanisms of MP removal depend on their structural and chemical properties, as shown in Figure 2 [50–52]. Many studies have been conducted to clarify the reaction pathways of these materials.

### Classification and potential of adsorbent nanomaterials

**Carbon-based adsorbents.** Carbon-based adsorbents, such as graphene oxide (GO), activated carbon, biochar, and carbon nanotubes (CNTs), have been extensively investigated regarding the treatment of pollutants in general and MPs in particular. By using corncob biochar, Abdoul Magid et al. showed an adsorption of polystyrene nanoplastics (PSNPs) of about 19 mg·g^−1^. The main mechanisms of PSNP adsorption include increased surface area from pyrolysis and oxidation, hydrophobic interactions (fresh biochar), hydrogen bonding through oxygen-containing groups (oxidized biochar), pore filling, and electrostatic interactions [53]. GO materials, such as a nickel/reduced graphene oxide (Ni/rGO) nanocomposite, also exhibited high adsorption efficiency, achieving 80.3% removal of PS from water containing 100 mg·L^−1^ PS. The primary mechanisms were hydrophobic and π–π interactions between PS microspheres and the Ni/rGO nanocomposite [54]. In addition, a mass loss of 35.66–50.46% of MP particles from aqueous polyethylene suspensions after 480 min was observed when using GO, GO-Cu_2_O, GO-MnO_2_, and GO-TiO_2_ for treatment [55]. Recently, Yan et al. developed a reduced graphene oxide (S-rGO) membrane with small lateral size and a rejection rate of up to 99.9% while maintaining high water permeability (236.2 L·m^−2^·h^−1^·bar^−1^) [56]. As another type of material belonging to carbon-based adsorbents, CNTs have also gained attention. Fabricated FeNi_12_-CNTs-800 samples achieved 100% removal of PVC after 20 min of treatment, with the mechanisms attributed to the hydrophobic surface and magnetic properties of the material [57]. In a water treatment plant, by applying granular activated carbon (GAC), Arenas et al. reached 90% of MP removal, and the main mechanism involved electrostatic attractions between the positive charge of MPs and negatively charge of GAC [58].

**Figure 3:**
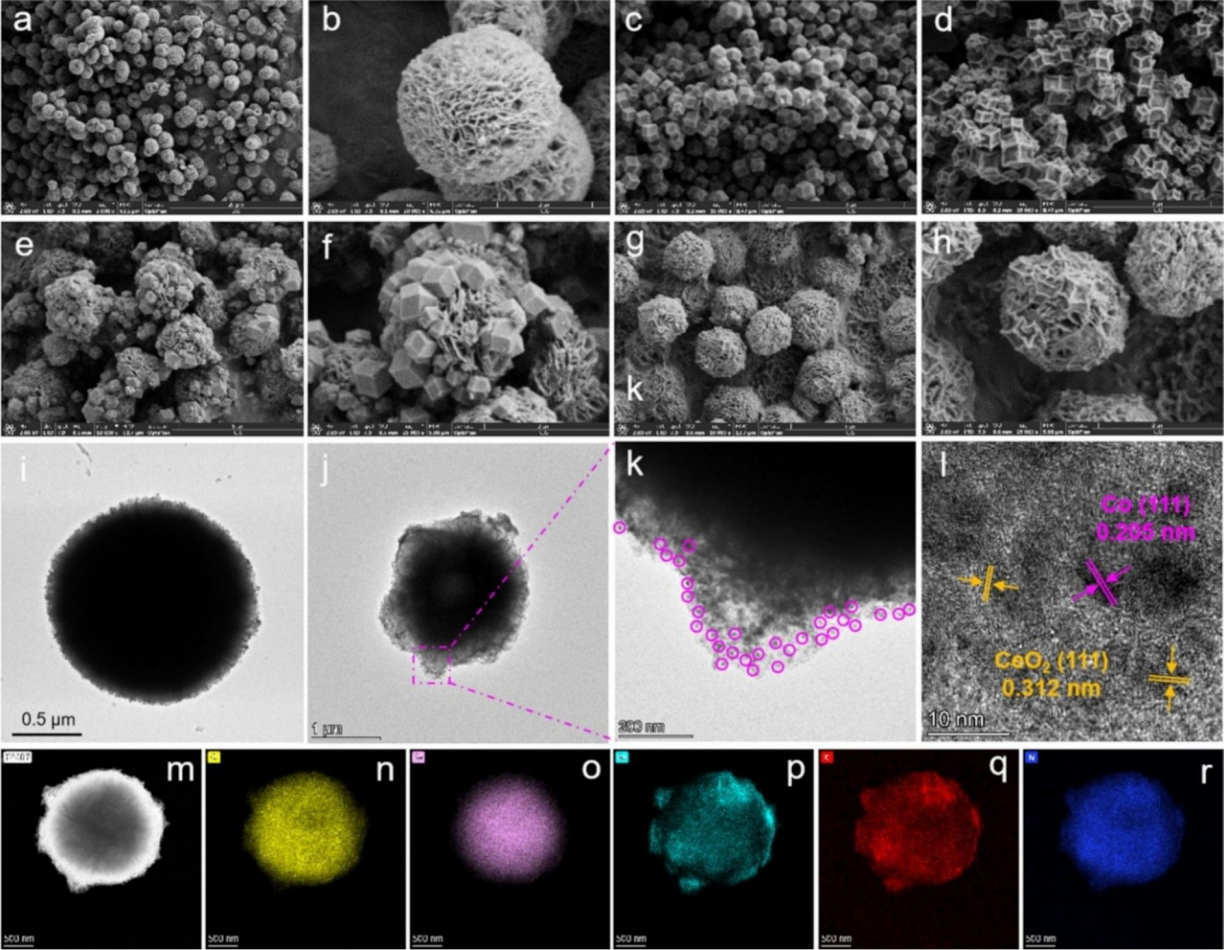
SEM and TEM image of synthesized Co-MOFs for removing MPs. SEM images of (a) CeO_2_, (b) CeO_2_ 3D flower-spheres, (c, d) ZIF-67 before and after calcination at 500 °C, (e, f) ZIF-67-90@CeO_2_, and (g, h) Co–N/C-90@CeO_2_ composites. TEM images of (i) CeO_2_ 3D flower-spheres, (j) Co–N/C-90@CeO_2_ composite. (k) Enlarged TEM image of Co–N/C-90@CeO_2_ composite. (l) HRTEM, (m) HAADF, and (n–r) elemental mapping images of Co–N/C-90@CeO_2_ composites [(n) O, (o) Co, (p) Ce, (q) C, and (r) N]. Figure 3 was reprinted from [67], *Chem. Eng. J.*, Vol. 514, by Wang, H.; Chen, H.; Wan, Q.; Zheng, Y.; Wan, Y.; Liu, X.; Song, X.; Ma, W.; Huo, P., “Catalytic degradation of polyethylene terephthalate microplastics by Co-N/C@CeO_2_ composite in thermal-assisted activation PMS system: Process mechanism and toxicological analysis”, Article No. 163192, Copyright (2025), with permission from Elsevier. This content is not subject to CC BY 4.0.

**Metal-organic frameworks.** MOFs are highly ordered crystalline materials characterized by high porosity and large surface area (Figure 3). Their pore size, volume, and functionality can be adjusted by changing the metal oxides and linkers, enabling easy synthesis and modification for diverse applications, including MP adsorption [59]. By using ZIF-8 nanocomposite, Pasanen et al. removed 99% of MPs after 1 h. The properties of nanocomposites showed highly porous structures (pore sizes of 0.3–3.4 nm) and adsorbed MPs through coordination and hydrophobic interactions [60]. Haris et al. introduced a magnetic C@FeO nanopillar adsorbent on a 2D-MOF, achieving approximately 100% removal of MPs (sizes <5 μm) after 1 h, significantly faster than conventional methods [61]. Similarly, You et al. reported on MOFs grown on a wood aerogel, ZIF-8@Aerogel, achieving removal efficiencies for polyvinylidene fluoride (PVDF) and PS particles of 91.4% and 85.8%, respectively [62]. Through electrostatic interactions, mesoporous UiO-66-NH_2_/P123 exhibited exceptional performance, achieving 100% removal of MPs from suspensions with an initial concentration of 1 g·L^−1^ [63]. In the study of Modak et al., a chromium-based metal-organic framework (Cr-MOF/MIL-101) was synthesized and achieved a significant PSNP removal efficiency of 96% from suspensions with initial concentrations of 5 and 70 ppm [64]. Based on the characteristics of MOFs, a MOF-covalent organic framework (COF) hybrid membrane (FS-50/COF(MATPA)-MOF(Zr)/PDA@PVDF) was constructed and achieved an MP removal rate of approximately 100%. This hybrid membrane was evaluated as a robust and environmentally friendly material [65]. Such hybrid materials show significant ability to remove MPs and should be further investigated to improve their properties and optimize operational parameters for practical application [66].

**Figure 4:**
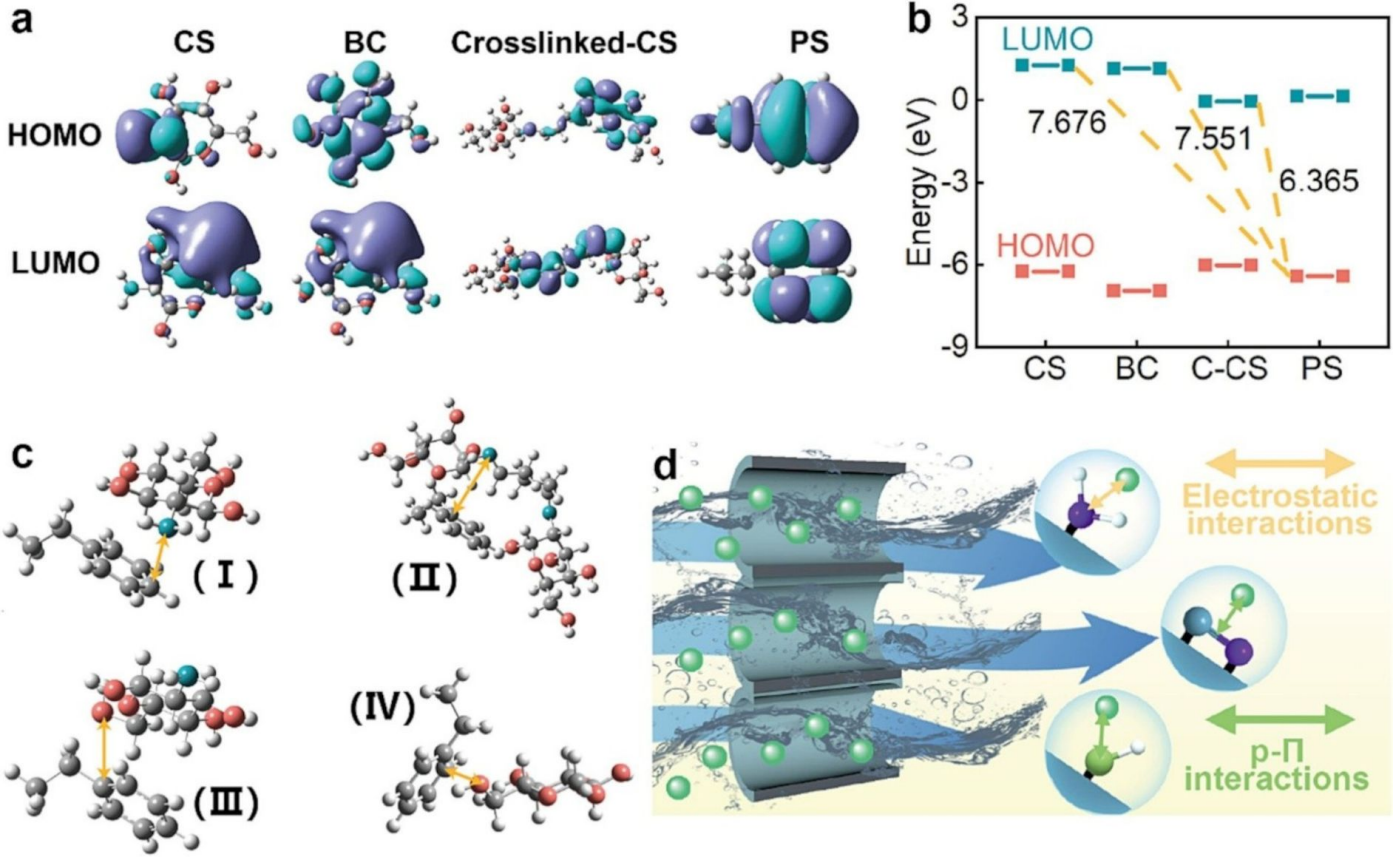
Illustration of (a) the highest occupied molecular orbital (HOMO) and lowest unoccupied molecular orbital (LUMO) of chitosan (CS), bacterial cellulose (BC), crosslinked CS, and PS. (b) Energy gaps among PS, CS, BC, and crosslinked-CS. (c) scenarios of hardwood vessel-inspired chitosan-based sponges (BGCS)-120 adsorbing PS. (d) Schematic diagram of adsorption mechanisms. Figure 4 was reprinted from [73], *Chem. Eng. J.*, Vol. 475, by Xu, J.; Guo, Y.; Tang, C.; Qian, Y.; Guo, C.; Wang, Z.; Li, L., “Hardwood vessel-inspired chitosan-based sponge with superior compressibility, superfast adsorption and remarkable recyclability for microplastics removal in water”, Article No. 146130, Copyright (2023), with permission from Elsevier. This content is not subject to CC BY 4.0.

**Aerogels and sponge-based adsorbents.** Porous materials, such as sponges and aerogels, can increase the number of adsorption sites for MPs/NPs, not only on the external surface but also within the internal pores, thereby enhancing the material’s adsorption rate (Figure 4) [49]. Chitin–graphene oxide sponges showed an adsorption capacity of 89.8% for PS. The defined mechanisms include electrostatic attraction between oppositely charged functional groups (amino and carboxyl groups), hydrogen bonding between oxygen-containing groups and amine or carboxyl groups, and π–π stacking between GO and the aromatic rings of MPs [68]. Similarly, obtaining an MP adsorption capacity above 90.4% after three cycles, Ko et al. also confirmed that the reaction mechanisms of MPs and graphene oxide–chitosan sponges were electrostatic interactions, hydrogen bonding, and π–π interactions through Fourier-transform infrared spectroscopy and X-ray photoelectron spectroscopy measurements [69]. Integrating different types of adsorbents such as the bimetallic organic framework (ZnCo-ZIF) and sponge, MS@ZnCo-ZIF@HDTMS was successfully fabricated and demonstrated an ability to remove over 98% of MPs through electrostatic forces and hydrogen bonding [70]. By fabricating bidirectionally ordered GO–nanocellulose aerogels (D-DPGG), Liu et al. demonstrated an adsorption efficiency above 80% over 20 adsorption cycles, attributed to electrostatic attraction and hydrogen bonding [71]. Recently, an eco-friendly lily bulb-derived polysaccharide aerogel was developed, demonstrating a significant removal efficiency of 93.68% for PS. The material also maintained a stable removal efficiency of over 90% during a 3 month evaluation period [72]. Based on the conducted studies, aerogels and sponge-based adsorbents, with their high adsorption efficiency and defined mechanisms, show great promise for the removal of MPs. The continued development of hybrid materials and eco-friendly options will provide more effective solutions for the elimination of MPs and other pollutants.

**Magnetic nanomaterials.** To optimize the efficiency of removing MPs, magnetic nanomaterials are often integrated with physical and chemical methods during the treatment process. According to Goel et al., the physical methods primarily rely on magnetic separation, while chemical approaches focus on nanoparticle functionalization to improve their effectiveness in microplastic removal [74]. Accordingly, numerous magnetic nanomaterials have been investigated and modified to improve functionalization. To be specific, by modifying the material, Wang et al. generated magnetic biochar, Mg-modified magnetic biochar, and Zn-modified magnetic biochar with PS removal efficiencies ranging from 94.81% to 99.46%. The created materials demonstrated good reusability, maintaining performance for six cycles with only a 5% efficiency loss, and enabled in situ degradation of MPs through thermal treatment to prevent desorption risks [75]. A high capture efficiency of microplastics was also achieved using novel magnetic composite nanoparticles composed of silica, gelatin, and chitosan. At a magnetic nanoseed concentration of just 0.002 g·L^−1^, these composites enabled 98% PET extraction, with the separation efficiency largely influenced by the morphology of the magnetic seeds. In addition to ensuring highly efficient magnetic separation of MP particles, this approach significantly reduced the volume of synthetic flocculant sludge [76]. By applying magnetic Fe_3_O_4_ nanoparticles, 83.1–92.9% of MPs with particle sizes ranging from 100 to 1000 nm were removed thanks to adsorption with magnetic separation [77]. These mechanisms are influenced significantly by factors like pH, ionic strength, and MP size, with sizes below 100 μm normally requiring optimized surface functionalization for effective treatment. The influence of these factors on removal efficiency has also been highlighted in numerous studies, particularly those focusing on the synthesis of magnetic nanomaterials [52,78].

### Comparison of adsorbent nanomaterials and other technologies

Water treatment processes play a crucial role in reducing MP concentrations in both water and wastewater. Conventional treatment plants, which typically employ coagulation, flocculation, sedimentation, filtration, and disinfection, can achieve a certain level of MP removal depending on the specific treatment processes and operational conditions. To be specific, when applying the coagulation/sedimentation and membrane filtration processes, the concentration of MPs decreased by about 49.6% in raw water. In a WTP, the conventional treatment process showed a removal efficiency of about 58.9–70.5% [79]. The efficiency of conventional processes is strongly affected by the size of MPs. Sedimentation has limited performance for small MPs (2–5 μm), achieving only 32.0 ± 4.5% removal [80]. Similarly, Han et al. reported a removal efficiency of 44.3 ± 3.4% using sedimentation, while Wang et al. observed 41–55% removal using Al_2_SO_4_ (25–32 ppm) [41,79]. Pivokonský et al. demonstrated that the coagulant type influences MP removal, with a maximum efficiency of 62% when using Al_2_SO_4_ [81]. Sand filtration is effective for larger MPs (>100 μm), but MPs in the range of 1–20 μm can easily pass through the filter layers, limiting the overall removal efficiency [41]. These observations highlight the persistence of MPs, especially those with small sizes, in treated water, revealing the limitations of current technologies and the need for ongoing innovation in water treatment.

Various technologies have been investigated for removing MPs, such as filtration [82], coagulation–flocculation [83], photocatalysis [84], and adsorbent nanomaterials [85]. Removal efficiencies vary widely, ranging from 47.1% for coagulation to over 90% for membrane bioreactors. Although achieving a high removal efficiency, the membrane bioreactor’s performance depends on different factors, including applied material, size, and surface area [86]. Due to the varying sizes, conventional membranes are often not suitable for removing MPs that have size scale fluctuations. In addition, the high cost and problems relating to membrane fouling can affect the filtration system performance [87]. Therefore, innovative and sustainable technologies to effectively remove MPs from various environments in general, and from water supply systems in particular, are crucial to be taken into account.

In this context, adsorbent nanomaterials, which possess high surface area and modified surface properties, have emerged as a promising technology for removing MPs from water supply systems [88]. In recent years, research focus on adsorbent nanomaterials has increased significantly. The differences in MP removal efficiency between conventional processes and adsorbent nanomaterials are summarized in Table 2, providing a clear comparison of technologies, efficiencies, and recent applications. Conventional processes exhibit moderate to high efficiencies, though their performance strongly depends on polymer type and particle size. In contrast, nanomaterials show higher and more significant removal efficiency, in some cases exceeding 99%. Particularly, multifunctional materials (e.g., Fe_3_O_4_@PDA-lipase nanoparticles or MOFs) combine adsorption, magnetic recovery, and even catalytic degradation, indicating their potential as next-generation solutions. By using coprecipitation and thermal decomposition, Aragón et al. synthesized magnetic nanoparticles to capture PE MPs. The results demonstrated that the thermal decomposition method achieved a capture efficiency of 69.3 ± 2.1% [89]. Modifying a cellulose nanofiber aerogel, Zhuang et al. showed the ability for MP removal with an improved adsorption capacity of 146.38 mg·g^−1^ for MPs [90]. Thus, while conventional methods remain practical and widely applied, nanomaterial-based strategies demonstrate superior effectiveness. Despite proving efficiency, potential nanomaterial leaching raises environmental concerns, leading to the necessity for risk assessments to ensure safe integration into water treatment processes.

**Table 2:** The effectiveness of various nanomaterials and conventional processes in removing MPs.

Treatment method	Chemical/material used	Target MP type (size)^a^	Removal efficiency	Ref.

Conventional methods				

coagulation–flocculation	Al_2_(SO_4_)_3_	PS (6–90 µm)	75.6–85.2%	[91]
coagulation	polyaluminium chloride	PP, PET, PVC, PA, ABS (150 μm)	37–56%	[92]
ultrafiltration	PVDF hollow fiber membrane	PP, PET, PVC, PA, ABS (150 μm)	≈100%	
coagulation	polyaluminium chloride, ferric chloride (FeCl_3_)	PS, PE (<500–5000 μm)	30.49–75.25%	[93]
coagulation-sedimentation	polyaluminium chloride, 1-methyl-3-propylimidazolium chloride, 1-decyl-3-methylimidazolium chloride	PS (0.1, 1, 10 μm)	≈97.2%	[94]
filtration	polycarbonate membrane, cellulose acetate membrane, polytetrafluoroethylene membrane	PA, PS (20–300 μm)	>94%	[86]
filtration	silica sand	PP, PS, PET, PVC (<10 μm)	84–98%	[95]
filtration	cellulose nanofiber-coated, delignified wood (CNF-CDW)	PS (25 μm)	95.97%	[96]

Nanomaterials				

carbon-based adsorbent	biochar-activated carbon, silica sand	PS, PA, PET (<20 μm)	81.24–96.26%	[97]
carbon-based adsorbent	banana peel biochar	PS (75–300 µm)	91.53–100%	[98]
carbon-based adsorbent	Ag-TiO_2_/carbon nanotubes	PS (1.94 μm)	31.7%	[99]
carbon-based adsorbent	GO-PVA-based membrane	HDPE (125 μm)	9%	[100]
magnetic nanoparticle	Fe_3_O_4_@PDA-lipase nanoparticles	PET (nanometer scale)	Fe_3_O_4_@PDA-lipase nanoparticles magnetically remove PET micro/nanoplastics	[101]
magnetic nanoparticle	magnetic pineapple waste activated carbon	PE, PS, PET (355 μm)	86.53–89.05%	[102]
aerogels-based adsorbent	taro stem microcrystalline cellulose aerogel	PS	92.37%	[103]
aerogels-based adsorbent	TCNF/FG aerogel	PE (6–10 μm)	93.3%	[104]
aerogels-based adsorbent	CNF/PVA/r-GO/Ga radial aerogels	microplastic	99.91%	[105]
biobased hydrogel adsorbent	bamboo powder PVA	PS, PE, PVC, PA (5 μm)	92.7–99.7%	[106]
metal-organic framework	Mn-doped ZnO	LDPE	85.4%	[107]
metal-organic framework	UIO-66-EDTMP (Zr-MOFs)	PS (100 nm)	97.45%	[108]
Metal-organic framework	Fe_3_O_4_@carboxymethyl-cellulose (CMC) - MOFs	PS, PP, PE, PVC, PET (3 μm)	98.0%	[109]

^a^Polyethylene (PE), polyethylene terephthalate (PET), polypropylene (PP), polyvinyl chloride (PVC), polyamide (PA), polystyrene (PS), acrylonitrile butadiene styrene (ABS), high-density polyethylene (HDPE), low-density polyethylene (LDPE), polymer-like polyvinyl alcohol (PVA), ethylenediamine tetramethylene phosphonic acid (EDTMP), TEMPO-oxidized cellulose nanofibers (TCNF), flash graphene (FG).

### Risk assessment of adsorbent nanomaterials

The use of adsorbent nanomaterials has shown promise for removing MPs from aqueous environments. However, their application may raise concerns about potential hazards, and the risks associated with these nanomaterials need to be considered. Nanomaterials can pose ecotoxicological risks because of their small size, high reactivity, and potential to persist in the environment. Specifically, applying magnetic nanoparticles for treatment purposes may release toxic metal ions (Ni or Mn) or highly soluble metal ions (Zn or Mg) into the treated water, which can then enter water bodies and cause harm [74]. Similarly, MOFs, such as materials combined with Fe, Cu, or Zr, may release toxic ions into water, affecting microbial communities and bioaccumulating in food chains, as shown in the study by Yang and colleagues [110]. Carbon-based nanomaterials, like GO, have the potential to react with biological systems, causing oxidative stress in aquatic organisms by generating reactive oxygen species, leading to cellular damage [111]. According to Thirunavukkarasu et al., the interactions between these nanomaterials and other contaminants are not fully understood. While some studies suggest that different synthesis methods can reduce toxicity, they still pose a significant threat to end-users [112]. Additionally, although certain nanomaterials are designed to be biodegradable, their actual degradation strongly depends on environmental conditions. In cases of incomplete or slow biodegradation, these materials may persist and accumulate in the environment. Thus, the production and disposal of nanomaterials may generate secondary pollutants, contributing to environmental contamination [49,113].

**Figure 5:**
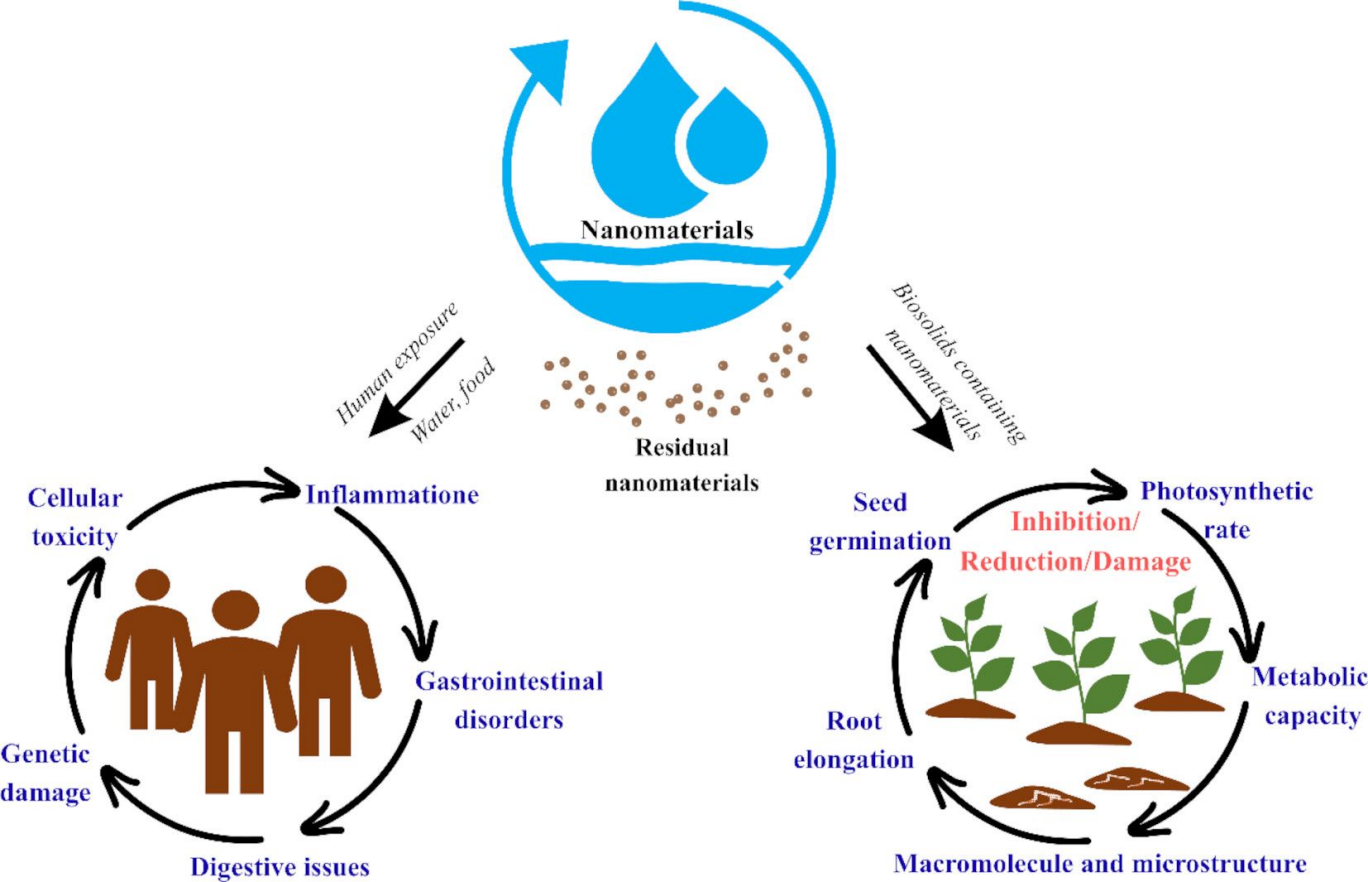
Adverse effects of residual nanomaterials.

Exposure to nanomaterials can occur through release into the environment during synthesis, application, or disposal [114]. Such exposure poses potential risks not only to human health, but also to the environment, where these materials can accumulate and interact with ecosystems, as illustrated in Figure 5. Aquatic organisms may be directly affected through water exposure, while humans can be exposed indirectly through the consumption of contaminated drinking water or seafood that has bioaccumulated these nanomaterials. Numerous adverse health effects, such as inflammation, gastrointestinal disorders, cellular toxicity, and genetic damage, are linked to nanomaterial accumulation, as shown in many studies [115]. Furthermore, the application of biosolids containing nanomaterials to agricultural soils can lead to their accumulation in the soil environment, potentially disrupting plant health and soil microbial communities, thereby affecting ecosystem sustainability [116]. Therefore, it is essential to develop safe nanomaterials and implement effective recovery strategies to minimize the release of unexpected materials into the environment.

To manage these risks, recovery methods like magnetic separation or filtration are important to reduce residual nanomaterials in treated water. Magnetic nanoparticles can be extracted from water using magnetic separation techniques [74]. Life cycle assessments (LCAs) are critical to evaluate the environmental footprint of nanomaterial production and disposal. According to Chakrapani et al., the LCAs are conducted in accordance with ISO 14040 standards. They cover relevant aspects, including raw material extraction, nanomaterial synthesis, application in water treatment, and decomposition at the end of use. The system boundaries are defined to capture the full life cycle, consisting of energy consumption, carbon emissions, and residual toxicity [113]. However, until now, in-depth LCAs conducted on nanomaterials are still relatively limited [117]. In addition, advances in anti-fouling technologies can enhance nanomaterial reusability, reducing costs and environmental impact. Although there are problems, such as the high cost of MOFs, their low stability in powder form, and the lack of standard tests for assessing nanomaterial toxicity [117]. Additionally, the long-term ecological impacts of nanomaterials remain understudied, particularly in soil environments.

### Future research directions and recommendations

Future research should prioritize the development of advanced adsorbent nanomaterials that align with sustainable development goals and support a circular economy in the long term. Nanomaterials with characteristics such as environmental durability and reusability should be considered to improve overall effectiveness. Many recent studies are focusing on porous and hybrid nanomaterials to enhance the efficiency and selectivity of MP removal in water treatment processes [70,72,118]. A comprehensive understanding of the adsorption mechanisms, including surface interactions and environmental factors like pH and ionic strength, is essential to optimize these nanomaterials. Additionally, scaling laboratory findings to real-world applications remains a challenge; thus, pilot-scale studies and field trials are crucial to assess the practicality, cost-effectiveness, and environmental impact of nanomaterials in various water treatment settings. Evaluating the potential toxicity, bioaccumulation, and environmental persistence of nanomaterials is vital to ensure their safe integration into existing water treatment infrastructures [119]. Furthermore, addressing challenges such as high production costs, scalability, and the risk of secondary pollution is imperative for the widespread adoption of nanomaterial-based technologies in MP elimination [120]. Finally, along with the development of information technology, the application of artificial intelligence (AI) and machine learning (ML) can transform water treatment. Leveraging pattern detection, ML simplifies MP classification and enhances nanomaterial identification, improving research efficiency and accuracy [121]. In addition, AI can aid in designing more efficient nanomaterials (zero-dimensional to three-dimensional) and predict their performance under varying environmental conditions. Thus, with AI support, the integration of nanomaterials into current water treatment systems can be optimized [122].

## Conclusion

MP pollution in water supply systems remains a pressing environmental and public health challenge because conventional treatment methods are unable to achieve complete removal, particularly for small particles. Adsorbent nanomaterials have shown strong potential for tackling this issue. Thanks to their ability to trap MPs through adsorption or magnetic separation, materials like carbon-based nanoparticles, magnetic particles, and MOFs have demonstrated removal efficiencies exceeding 90%. Their tunable properties, reusability, and potential for multifunctional performance position them as promising next-generation materials for mitigating MP contamination. However, the translation of laboratory successes to large-scale water treatment remains constrained by critical challenges, including potential nanomaterial toxicity, high production costs, limited stability, and the risk of secondary pollution. Standardized testing protocols, comprehensive LCAs, and advanced recovery strategies are urgently needed to ensure safe and sustainable deployment. Moreover, detecting and removing sub-micrometer plastics (<1 μm), which pose significant health risks due to their ability to penetrate tissues, continues to be a major obstacle. For further investigation, the development of safe, affordable, and environmentally benign nanomaterials, which are integrated with smart treatment systems, could transform current water treatment infrastructures. By combining scientific innovation with practical scalability, adsorbent nanomaterials offer a strong foundation for sustainable solutions that safeguard both human health and aquatic ecosystems.

## Data Availability

All data that supports the findings of this study is available in the published article and/or the supporting information of this article.
